# Phase I/II study of the deacetylase inhibitor panobinostat after allogeneic stem cell transplantation in patients with high-risk MDS or AML (PANOBEST trial)

**DOI:** 10.1038/leu.2017.242

**Published:** 2017-09-01

**Authors:** G Bug, A Burchert, E-M Wagner, N Kröger, T Berg, S Güller, S K Metzelder, A Wolf, S Hünecke, P Bader, J Schetelig, H Serve, O G Ottmann

**Affiliations:** 1 Department of Medicine II, University Hospital, Frankfurt, Germany; 2 Department of Hematology, Oncology and Immunology, Universitätsklinikum, Marburg, Germany; 3 Third Department of Medicine, University Medical Center, Mainz, Germany; 4 Department of Stem Cell Transplantation, University Hospital, Hamburg, Germany; 5 Department for Children and Adolescents, University Hospital, Frankfurt, Germany; 6 Medizinische Klinik und Poliklinik I, Universitätsklinikum, Dresden, Germany; 7 Division of Cancer and Genetics, Department of Haematology, Cardiff University, Cardiff, UK

Maintenance therapy after allogeneic hematopoietic stem cell transplantation (HSCT) for acute myeloid leukemia (AML) or myelodysplastic syndrome (MDS) is conceptually attractive to prevent relapse, but has been hampered by the limited number of suitable anti-leukemic agents. The deacetylase inhibitor (DACi) panobinostat demonstrated moderate anti-leukemic activity in a small subset of patients with advanced AML and high-risk MDS in phase I/II trials.^1, 2^ It also displays immunomodulatory activity^3^ that may enhance leukemia-specific cytotoxicity^4^ and mitigate graft versus host disease (GvHD), but conversely could impair T- and NK cell function.^5, 6^ We conducted this open-label, multi-center phase I/II trial (NCT01451268) to assess the feasibility and preliminary efficacy of prolonged prophylactic administration of panobinostat after HSCT for AML or MDS. The study protocol was approved by an independent ethics committee and conducted in compliance with the Declaration of Helsinki. All patients provided written informed consent.

Patient eligibility and study design are summarized in Supplementary Figure S1. Briefly, between January 2011 and January 2015, 42 patients (37 AML, 5 MDS) were enrolled at a median of 96 days (60–147) post HSCT. Patients had to be in complete hematologic remission (CR) post HSCT, and fulfill one or more of the following criteria: (i) AML refractory or with delayed response to or relapsed after greater than or equal to one cycle of standard chemotherapy; (ii) adverse risk cytogenetics; (iii) secondary to MDS or radio/chemotherapy; (iv) MDS intermediate-2 or high risk according to international prognostic scoring system or MDS refractory anemia with excess blasts (WHO classification). At transplant, 67% of patients (*n*=28) had active disease (bone marrow blasts 8–80%, median 21%, 1 patient with isolated extramedullary AML), 9 were in CR1 (21%) and 5 in CR2 (12%).

Primary objective of the phase I part was determination of the maximum tolerated dose (MTD) and dose-limiting toxicity (DLT) of panobinostat, given orally thrice weekly (TIW) in one of two sequentially tested administration schedules: weekly (schedule A; starting dose 10 mg) or every other week (schedule B; starting dose 20 mg) using a 3+3 design. DLT was determined separately in both schedules during the first 28 days of panobinostat treatment, which was scheduled for up to 1 year. In phase 2, patients were randomized 1:1 to schedule A or B at the respective MTD.

Patient and transplant characteristics were equally distributed between both schedules (Supplementary Table S1). Median age was 52 (21–71) years and eastern cooperative group performance status either 0 (57%) or 1 (43%). Patient disposition is outlined in Supplementary Figure S2. All 12 patients in the phase 1 part of schedule A and 11 of 12 patients in schedule B were evaluable for MTD. Five DLTs were observed, three in schedule A (fatigue G3 at 20 mg, colitis and nausea/emesis G3 at 30 mg in one patient each) and two in schedule B (diarrhea and headache G3 at 40 mg in one patient each). One patient discontinued study treatment after three doses of panobinostat (schedule B, 20 mg TIW) because of G2 electrocardiogram alterations; this patient was not evaluable for DLT and was replaced. The MTDs for schedules A (weekly) and B (every other week) were 20 mg and 30 mg TIW, respectively, and were selected as recommended phase 2 dose. These MTDs resemble those in patients with myeloma requiring prolonged therapy.^7^

All patients were analyzed for safety. Thirty-five of 42 patients (83%) experienced at least one G3/4 adverse event (AE), considered panobinostat-related in 22 patients (52%). Rates of G3/4 AEs did not differ significantly between schedules (A: *n*=12, 57% B: *n*=10, 48%). All panobinostat-related G3/4 AEs and G1/2 AEs that constituted DLTs or triggered dose reductions are listed in Table 1. Panobinostat-related AEs were fully and rapidly reversible after interrupting panobinostat. Thrombocytopenia G3/4 was observed in both treatment schedules (A: *n*=6, 28% B: *n*=4, 19%); in schedule B, platelet counts recovered to baseline values by day 15. Clinically relevant constitutional symptoms were observed only with schedule A. No patient died on treatment or within 28 days of the last panobinostat dose, and 10 patients died post study (relapse *n*=6, sepsis *n*=1, severe chronic GvHD *n*=1, relapse of pre-existing lung cancer *n*=1 and sudden death 3.5 months after study discontinuation *n*=1).

The most common G3/4 AEs irrespective of causality are listed in Supplementary Table S2. Twelve patients (29%) developed AEs that led to permanent discontinuation of panobinostat after a median of 30 days (7–293). Previous studies showed that tolerability and hematologic AEs differed by schedule of panobinostat administration and that the MTD was not necessarily compatible with prolonged administration.^2, 8, 9^ In our study, 22 patients (52%) received panobinostat for 1 year as scheduled (A: 10/21 patients, 52 vs B: 12/21, 57%), and seven of these (17%) required no dose interruptions or reductions. Reasons for premature discontinuation of study drug were AEs (*n*=12), relapse (*n*=5), patient decision (*n*=2) or prohibited co-medication (*n*=1). Of 27 patients treated at the MTD, 15 (55%) discontinued early after a median of 47 days (11–172); additional 4 patients (15%) required dose reductions. Median duration of treatment at the MTD was 52 days (range, 11–368) in schedule A versus 228 days (16–365) in schedule B (*P*=0.34). Alternating week administration resulted in delivery of a higher cumulative dose at the MTD and was more compatible with long-term administration. In comparison, in studies of post-transplant maintenance with hypomethylating agent, only 20–43% of patients received all scheduled cycles of hypomethylating agent (summarized in Brunner *et al.*^10^).

Donor lymphocyte infusions (DLIs) were permitted by the study protocol at the discretion of the treating physician. Eighteen patients (43% in schedule A and B) received a median of two DLIs (1–6), and initiated a median of 88 days (49–317) and 104 days (50–231) after the first panobinostat dose (median 0.2 × 10^6^ and 0.9 × 10^6^ CD3^+^ cells per kg body weight, respectively). Only 4/42 patients developed acute GvHD on study (G1, *n*=1; G3, *n*=3), all in schedule A. Of note, the proportion of regulatory T cells decreased with schedule A, while remaining stable in schedule B (Supplementary Figure S3). Cumulative incidence of moderate (*n*=10) or severe (*n*=2) chronic GvHD was 29% (95% confidence interval (CI), 16–42%) at 2 years after starting panobinostat and did not differ between schedules.

At 2 years after the first panobinostat dose, the cumulative incidence of relapse and non-relapse mortality across all dose levels was 20% (95% CI, 7–33%) and 5% (95% CI, 0–11%, Figure 1a). Thus, the low relapse rate observed in the PANOBEST trial was not offset by a higher than expected incidence of clinically significant chronic GvHD even among patients receiving additional DLI, suggesting that panobinostat does not impair development of peripheral tolerance and may actually mitigate GvHD. These data are consistent with a phase I/II study of short-term peri-transplant vorinostat showing a significantly lower incidence of acute GvHD greater than or equal to G2 compared to historical controls.^11^

To date, median overall survival (OS) and relapse free survival have not been reached after a median follow-up of 22 months (range, 6–57) (Figures 1b and c). Probabilities of 2-year OS and relapse free survival are 81% (95% CI, 69–95%) and 75% (95% CI, 63–90%), respectively. In view of the median time to relapse (4–6 months after HSCT), the time from HSCT to starting panobinostat (median 96 days, 60–147) may have introduced a positive selection bias and led to under-representation of patients with very aggressive AML. Nevertheless, outcome in our high-risk AML and MDS population compares favorably with survival rates and cumulative incidence of relapse (exceeding 30–60% at 2–3 years) reported for similar patient cohorts.^12, 13, 14, 15^ The definite role of panobinostat maintenance after HSCT for high-risk myeloid malignancies will be determined in a large European randomized trial, using the alternate week dosing regimen found to be better tolerated in the present trial.

## Figures and Tables

**Figure 1:**
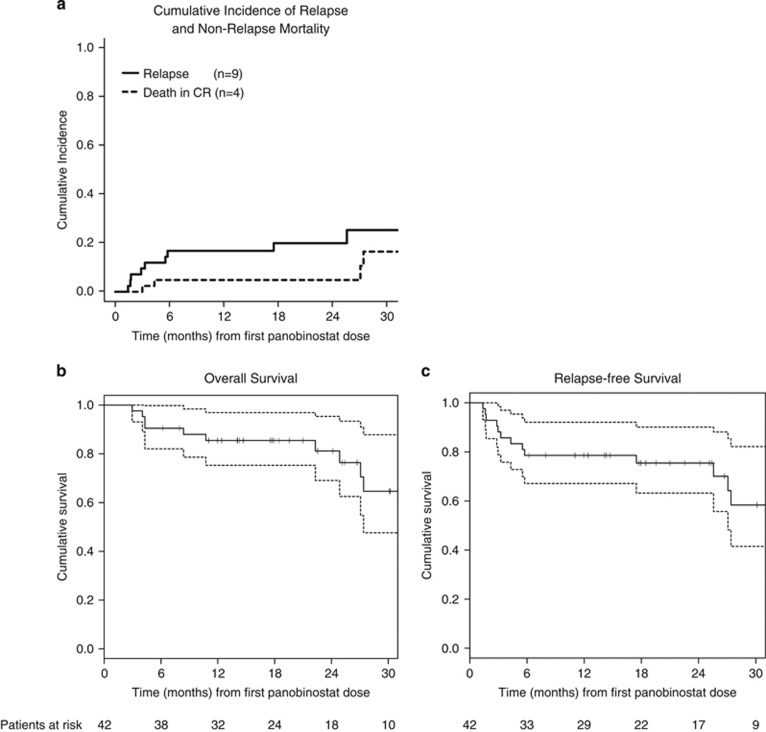
(**a**) Cumulative incidence of relapse and non-relapse mortality. (**b**) Overall survival. (**c**) Relapse-free survival. Kaplan–Meier curves are shown for all patients enrolled and calculated from the first dose of panobinostat. Symbols represent censoring times.

**Table 1:** Adverse events considered related to panobinostat by treatment schedule and initial dose cohort

*Panobinostat-related toxicity*	*Arm A,* n *(%)*			*Arm B,* n *(%)*		
	*G1 and 2*	*G3*	*G4*	*G1 and 2*	*G3*	*G4*
*Blood/bone marrow*						
Leukocytopenia	0	2 (10)	0	0	2 (10)	0
Neutropenia	0	1 (5)	1 (5)	0	4 (19)	0
Thrombocytopenia	0	5 (24)	1 (5)	0	3 (14)	1 (5)
Anemia	0	2 (10)	0	0	0	0
						
Cardiac	1 (5)	0	0	1 (5)	0	0
						
*Constitutional symptoms*						
Fatigue	2 (10)	4 (19)	0	0	0	0
Weight loss	1 (5)	0	0	0	0	0
						
*Gastrointestinal symptoms*						
Nausea/vomiting	4 (19)	1 (5)	0	3 (14)	0	0
Diarrhea	3 (14)	1 (5)	0	1 (5)	2 (10)	0
Colitis	0	1 (5)	0	0	0	0
Anorexia	1 (5)	0	0	0	0	0
Oral mucositis	0	0	0	1 (5)	0	0
Taste alteration	0	0	0	1 (5)	0	0
						
Pain	1 (5)	1 (5)	0	1 (5)	0	0
Headache	0	0	0	0	1 (5)	0
						
Renal failure	0	1 (5)	0	0	0	0
Rash	0	0	0	1 (5)	0	0
Sensory neuropathy	0	0	0	0	1 (5)	0
						
*Metabolic/laboratory*						
Elevated liver function tests	0	0	0	2 (10)	2 (10)	0
Creatinine increased	1 (5)	0	0	0	0	0
Hyperuricemia	0	1 (5)	0	0	0	0
