# Supplementary material for: Phase I/II study of the deacetylase inhibitor panobinostat after allogeneic stem cell transplantation in patients with high-risk MDS or AML (PANOBEST trial)
Source: Leukemia. 2017 Sep 1;31(11):2523–5. doi: 10.1038/leu.2017.242 (PMC5668491; doi:10.1038/leu.2017.242)
Supplement: Supplementary Table S1 [file leu2017242x1.doc]

**Table S1: Patient, disease and HSCT characteristics**

|  | **Schedule A**  **(N=21)** | **Schedule B**  **(N=21)** | **P Value** |
| --- | --- | --- | --- |
| **Median age**, years (range) | 58 (21-68) | 50 (30-71) | ns |
| **ECOG performance status**  0  1 | 10 (48%)  11 (52%) | 14 (67%)  7 (33%) | ns |
| **Sex**  male / female | 10 (48%) / 11 (52%) | 14 (67%) / (33%) | ns |
| **Disease**  AML / MDS | 19 (90%) / 2 (10%) | 18 (86%) / 3 (14%) | ns |
| de novo  secondary to MDS  therapy-associated | 15 (72%)  3 (14%)  3 (14%) | 16 (76%)  2 (10%)  3 (14%) |  |
| **Karyotype (ELN criteria), AML only (n=37)**  good  intermediate-1/-2  adverse | 2 (10%)  10 (53%)  7 (37%) | 3 (17%)  9 (50%)  6 (33%) | ns |
| **Stage prior to HSCT**  CR1/CR2  primary refractory  relapse  untreated | 6 (28%)  10 (48%)  2 (10%)  3 (14%) | 8 (38%)  7 (33%)  5 (24%)  1 (5%) | ns |
| **Median BM blasts at start of conditioning** (range), n=28 | n=15  20 (8-63) | n=13  23 (0-80%) | ns |
| **Donor**  MRD / MMRD  MUD / MMUD | 4 (19%) / 2 (10%)  13 (61%) / 2 (10%) | 5 (24%) / 1 (5%)  12 (57%) / 3 (14%) | ns |
| **Conditioning regimens**  Myeloablative (MAC)  Reduced-intensity (RIC)  Fludarabine/Melphalan +/- BCNU  Flamsa-RIC  Fludarabine/Melphalan/TBI 2-8 Gy  Fludarabine/TBI 2-8 Gy  Fludarabine/Busulfan  Other | 4 (19%)  17 (81%)  7  5  3  2  1  3 | 3 (14%)  18 (86%)  7  3  3  3  2  3 | ns  % of all patients  34%  19%  14%  12%  7%  14% |
| **Immunosuppressive medications at enrolment**  None  Ciclosporin A  additional mycophenolate mofetil  additional prednisolone | 3 (14%)  18 (86%)  2  5 | 6 (29%)  15 (71%)  1  3 | ns |
| **CD34^+^ subset chimerism at enrolment**  Complete donor chimerism  Mixed chimerism  Not available | 5 (24%)  9 (43%)  7 (33%) | 6 (29%)  9 (42%)  6 (29%) | ns |
| **GvHD at enrolment**  None  Acute GvHD (grade 1)  Chronic GvHD (mild) | 18 (86%)  2 (9%)  1 (5%) | 12 (57%)  3 (14%)  6 (29%) | ns |
| **Infections at enrolment**  None  CMV reactivation  Herpes stomatitis  Polyoma virus cystitis  Urinary tract infection | 16 (76%)  3 (14%)  1 (5%)  1 (5%)  0 | 16 (76%)  3 (14%)  1 (5%)  0  1 (5%) | ns |

Abbreviations: ELN, European Leukemia Net; HSCT, hematopoietic stem cell transplantation; BM, bone marrow; MRD, matched related donor; MMRD, mismatched related donor; MUD, matched unrelated donor; MMUD, mismatched unrelated donor; Flamsa-RIC, fludarabine, amsacrine, AraC, reduced-intensity conditioning; TBI, total body irradiation; Gy, Gray;
