# Supplementary material for: Phase I/II study of the deacetylase inhibitor panobinostat after allogeneic stem cell transplantation in patients with high-risk MDS or AML (PANOBEST trial)
Source: Leukemia. 2017 Sep 1;31(11):2523–5. doi: 10.1038/leu.2017.242 (PMC5668491; doi:10.1038/leu.2017.242)
Supplement: Supplementary Table S2 [file leu2017242x2.docx]

| **AEs regardless of causality** | **Schedule A, n (%)** | | **Schedule B, n (%)** | |
| --- | --- | --- | --- | --- |
|  | **Grade 3** | **Grade 4** | **Grade 3** | **Grade 4** |
| Blood/Bone marrow | 6 (29) | 5 (24) | 9 (43) | 1 (5) |
| Cardiac | 0 | 1 (5) | 0 | 0 |
| Constitutional symptoms  Fatigue  Reduced general condition  Fever | 4 (19)  1 (5)  0 | 0  0  0 | 3 (14)  0  3 (14) | 0  0  0 |
| Neurology | 2 (10) | 0 | 1 (5) | 0 |
| Infection | 5 (24) | 0 | 5 (24) | 0 |
| Gastrointestinal symptoms  Nausea/Vomiting  Diarrhea  Colitis  Oral mucositis  Fistula: anus | 1 (5)  2 (10)  1 (5)  1 (5)  0 | 0  0  0  0  0 | 0  2 (10)  0  0  1 (5) | 0  0  0  0  0 |
| Pain  Headache | 1 (5)  0 | 0  0 | 0  1 (5) | 0  0 |
| Renal failure | 1 (5) | 0 | 0 | 0 |
| cGvHD liver | 1 (5) | 0 | 0 | 0 |
| Pulmonary fibrosis | 0 | 0 | 1 (5) | 0 |
| Relapse NSCLC | 0 | 0 | 0 | 1 (5) |
| Thrombosis | 0 | 1 (5) | 0 | 0 |
| Metabolic/Laboratory  Elevated liver function tests  Hyperuricemia  Diabetes  Hyperkalemia  Hypokalemia  Hypocalcemia  Hypertriglyceridemia  Amylase/Lipase | 1 (5)  2 (10)  1 (5)  1 (5)  0  1 (5)  0  1 (5) | 0  0  0  0  0  0  0  1 (5) | 3 (14)  0  0  1 (5)  1 (5)  0  1 (5)  1 (5) | 1 (5)  0  0  0  0  0  0  2 (10) |

**Table S2:** Adverse events grade 3 and 4 regardless of causality to study drug
