# Supplementary material for: Phase I/II study of the deacetylase inhibitor panobinostat after allogeneic stem cell transplantation in patients with high-risk MDS or AML (PANOBEST trial)
Source: Leukemia. 2017 Sep 1;31(11):2523–5. doi: 10.1038/leu.2017.242 (PMC5668491; doi:10.1038/leu.2017.242)
Supplement: Supplementary Figure S1 [file leu2017242x3.ppt]

## Slide 1
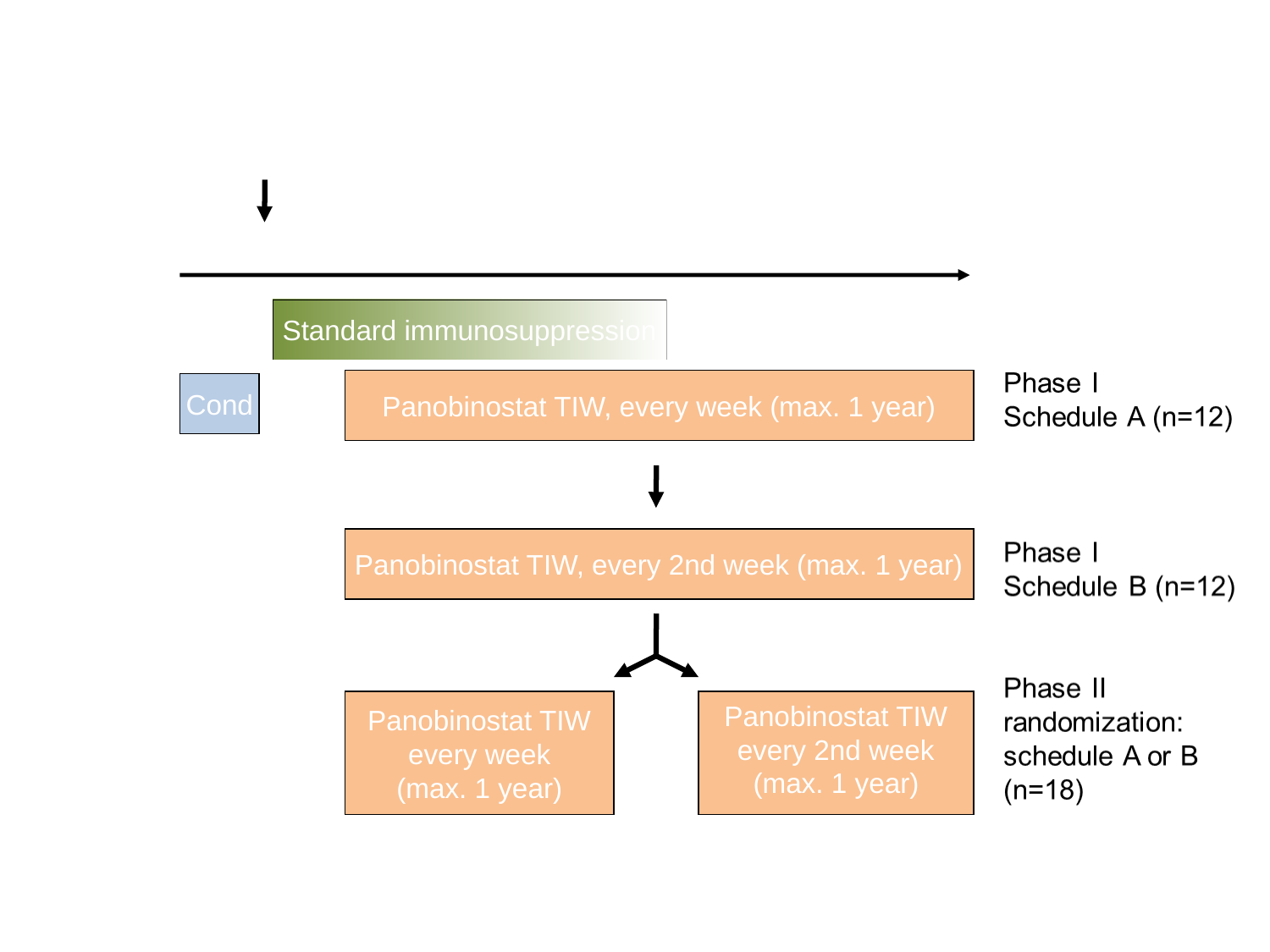

Figure S1
HSCT
Days
after HSCT 0 ≥ +60
Standard immunosuppression
Panobinostat TIW, every week (max. 1 year)
Cond
Panobinostat TIW, every 2nd week (max. 1 year)
R
Panobinostat TIW
every week
(max. 1 year)
Panobinostat TIW
every 2nd week
(max. 1 year)
#
