# Supplementary material for: Phase I/II study of the deacetylase inhibitor panobinostat after allogeneic stem cell transplantation in patients with high-risk MDS or AML (PANOBEST trial)
Source: Leukemia. 2017 Sep 1;31(11):2523–5. doi: 10.1038/leu.2017.242 (PMC5668491; doi:10.1038/leu.2017.242)
Supplement: Supplementary Figure S2 [file leu2017242x4.ppt]

## Slide 1
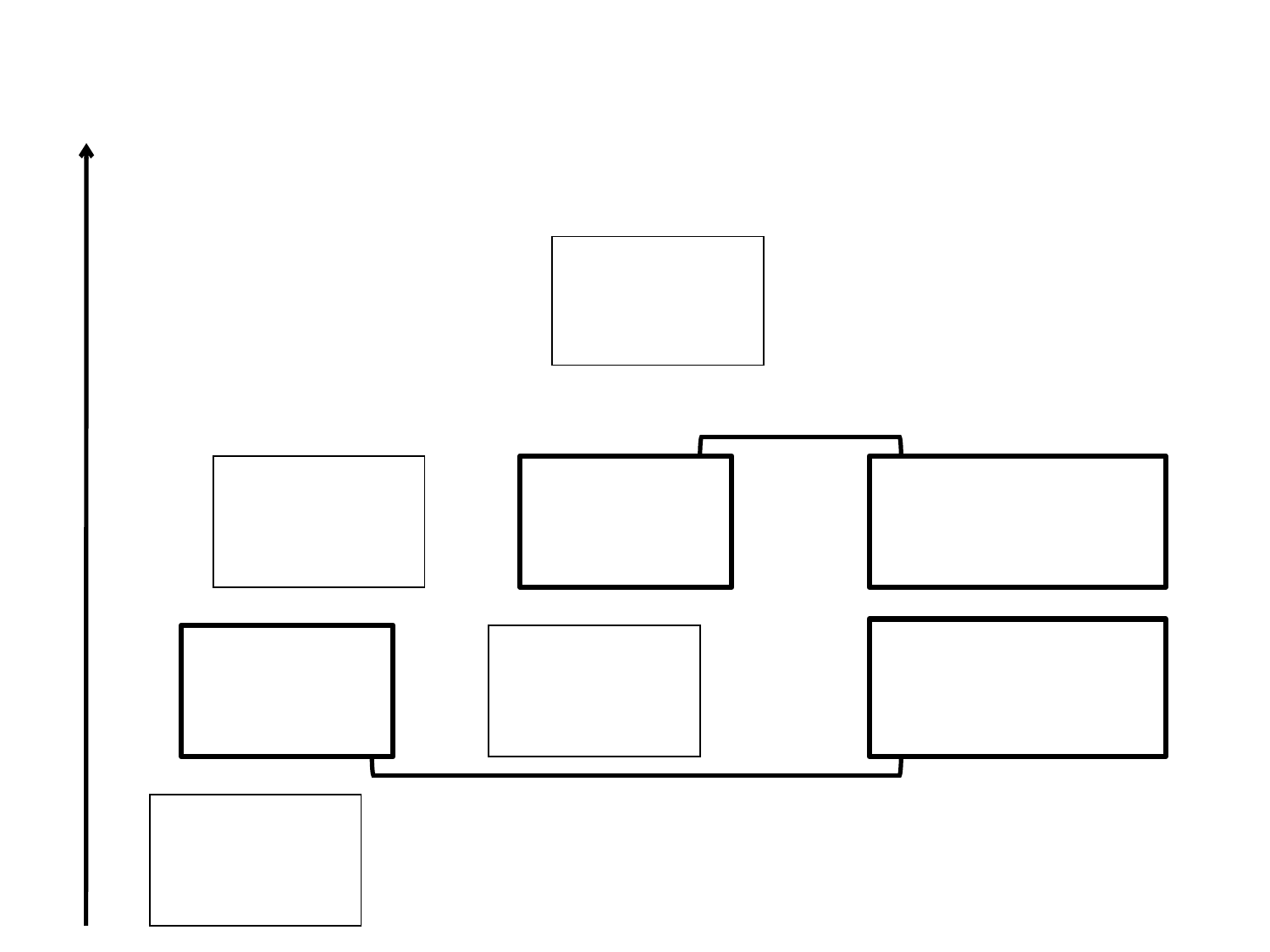

Figure S2
Dose-escalation (Phase I, n=24)
Expansion (Phase II, n=18)
Schedule A (n=12)
PAN every week
Schedule B (n=12)
PAN every other week
Cohort 6
(n=5/5)
PAN 40 mg
MTD, schedule B
Cohort 3
(n=3/3)
PAN 30 mg TIW
Cohort 5
(n=3/3)
PAN 30 mg TIW
Cohort 7B (n=9)
PAN 30 mg TIW
every other week
Panobinostat dose levels
Cohort 2
(n=6/6)
PAN 20 mg TIW
Cohort 4
(n=3/4)
PAN 20 mg TIW
Cohort 7A (n=9)
PAN 20 mg TIW
every week
MTD, schedule A
Cohort 1
(n=3/3)
PAN 10 mg TIW
