# Supplementary figures and images for: Phase I/II study of the deacetylase inhibitor panobinostat after allogeneic stem cell transplantation in patients with high-risk MDS or AML (PANOBEST trial)
Source: Leukemia. 2017 Sep 1;31(11):2523–5. doi: 10.1038/leu.2017.242 (PMC5668491; doi:10.1038/leu.2017.242)

## Slide 1
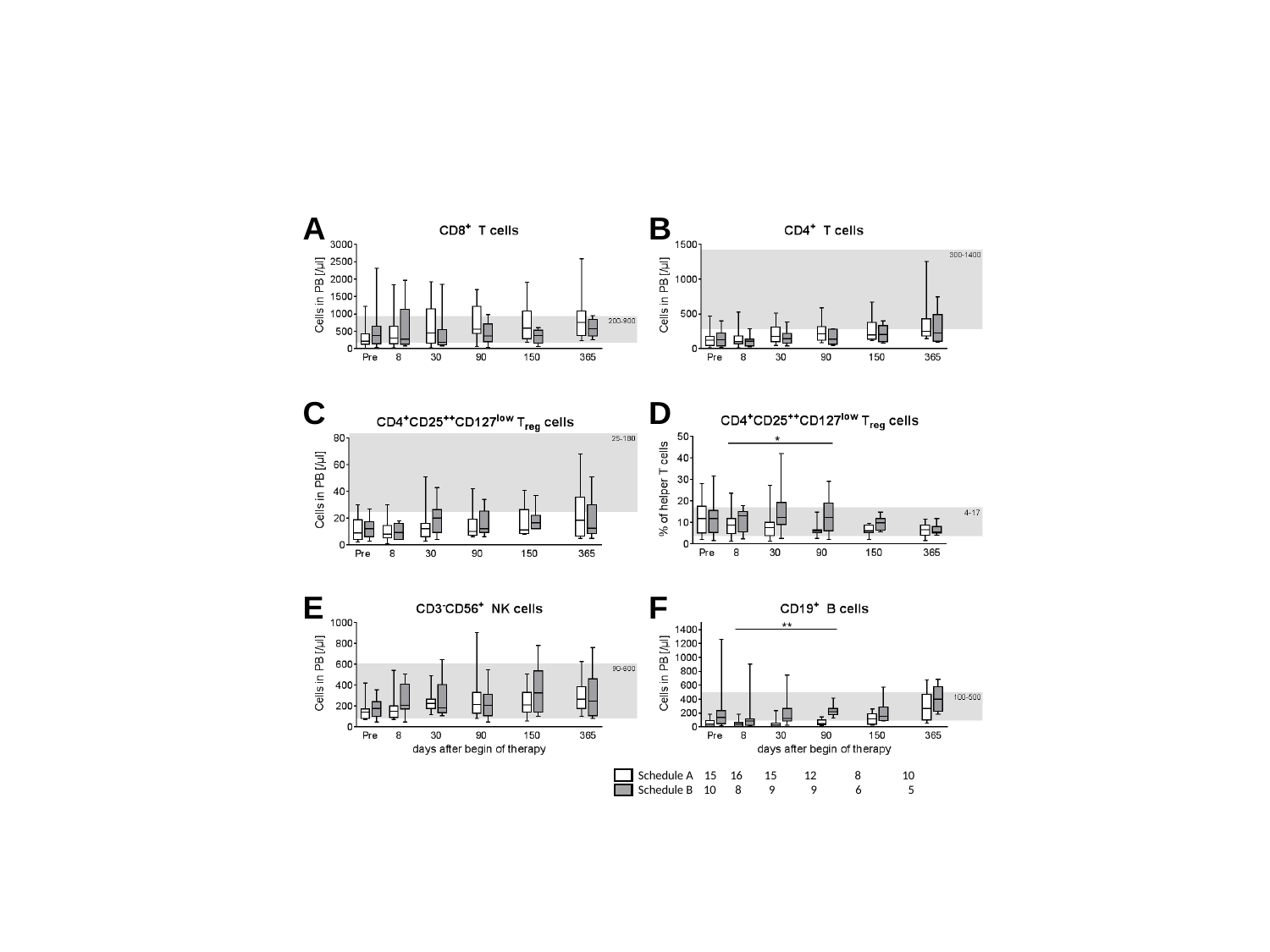

Figure S2
A
B
C
D
E
F
Schedule A 15 16 15 12 8 10
Schedule B 10 8 9 9 6 5

Supplement: Supplementary Figure S3 [file leu2017242x5.ppt]
