# Supplementary material for: Phase I/II study of the deacetylase inhibitor panobinostat after allogeneic stem cell transplantation in patients with high-risk MDS or AML (PANOBEST trial)
Source: Leukemia. 2017 Sep 1;31(11):2523–5. doi: 10.1038/leu.2017.242 (PMC5668491; doi:10.1038/leu.2017.242)
Supplement: Supplementary Figure Legend S1 [file leu2017242x6.docx]

**Figure legend S1**:

**Study design**. In phase 1, patients received escalating doses of panobinostat, initially administered three times per week (TIW) every week (Schedule A), a subsequent patient cohort received panobinostat in alternating weeks (Schedule B). In phase 2, patients were randomized to receive treatment with panobinostat either according to schedule A or schedule B, at the respective maximum tolerated dose (MTD) determined for each schedule. Time window for enrollment was 60 to 150 days after transplant. Cond, conditioning; HSCT, hematopoietic stem cell transplantation

**Patient eligibility**

Adult patients with high-risk AML (except acute promyelocytic leukemia) or MDS intermediate-2 or high-risk according to the International Prognostic Scoring System (IPSS), or RAEB according to the World Health Organization (WHO) classification in complete hematologic remission post-HSCT were eligible. High-risk AML patients had to fulfill one or more of the following criteria: (i) refractory to or relapsed after at least one cycle of standard chemotherapy; (ii) >10% bone marrow blasts at day 15 of the first induction cycle; (iii) adverse risk cytogenetics including complex karyotype (≥3 abnormalities or abnormalities of chromosomes 3, 5 or 7) regardless of stage; (iv) secondary to MDS or radio-/chemotherapy. All conditioning regimens except for >8 Gy total body irradiation or >9.6 mg/kg total i.v. busulfan dose were permitted. Patients had to be enrolled between days 60-150 after HSCT. Key inclusion criteria included Eastern Cooperative Oncology Group performance status (ECOG PS) ≤2, ANC ≥1,000/μL, platelets ≥75,000/μL, adequate organ function and no active acute GvHD ≥ grade 2 or extensive chronic GvHD. Patients with clinical symptoms of central nervous system leukemia were excluded. Additionally, patients receiving a drug known to prolong the QT time (i.e. tacrolimus, fluconazole, posaconazole, foscarnet, macrolide antibiotics, moxifloxacin) or who had received prior treatment with a DACi were not eligible.

**Safety and Efficacy Assessments**

Patients were monitored for safety throughout the trial and up to 28 days after the last dose of study treatment. AEs were assessed according to the Common Terminology Criteria for Adverse Events (CTCAE) version 3.0. DLT was defined as prolonged grade 4 (G4) hematologic toxicity or any non-hematologic toxicity ≥G3 unrelated to disease progression or intercurrent illness within 28 days of the first panobinostat dose. In addition, acute GvHD ≥G3 scored with modified Gluckberg criteria (1) or moderate or severe chronic GvHD as assessed according to the National Institutes of Health consensus criteria (2) were also regarded as DLT. Hematopoietic chimerism was assayed in the peripheral blood as previously described (3), (4)

**Statistical analysis**

Patients were evaluated on an intent-to-treat basis and all patients who received at least one dose of study drug were included in the analysis. Overall survival (OS) and relapse-free survival (RFS) probabilities were estimated with the Kaplan-Meier method. Time was measured from the day of the first dose of panobinostat until death or last follow-up. Events for RFS were death or hematologic relapse. The cumulative incidences of relapse, non-relapse mortality (NRM) and chronic GvHD were estimated using competing events statistics. Proportions were compared by means of the χ^2^ method, whereas the Mann-Whitney test or paired samples t-test was applied to detect differences between continuous parameters. IBM SPSS statistics version 23 and R version 3.2.1 were used as statistical software.

Objectives of phase 2 were to generate preliminary efficacy data of panobinostat at the MTD. As patient enrollment was permitted until day 150 after HSCT, outcome measures reported refer to the first day of panobinostat dosing.

References:

1. Przepiorka D, Weisdorf D, Martin P, Klingemann HG, Beatty P, Hows J, et al. 1994 Consensus Conference on Acute GVHD Grading. Bone marrow transplantation. 1995;15(6):825-8.

2. Filipovich AH, Weisdorf D, Pavletic S, Socie G, Wingard JR, Lee SJ, et al. National Institutes of Health consensus development project on criteria for clinical trials in chronic graft-versus-host disease: I. Diagnosis and staging working group report. Biol Blood Marrow Transplant. 2005;11(12):945-56.

3. Willasch A, Eing S, Weber G, Kuci S, Schneider G, Soerensen J, et al. Enrichment of cell subpopulations applying automated MACS technique: purity, recovery and applicability for PCR-based chimerism analysis. Bone marrow transplantation. 2010;45(1):181-9.

4. Bader P, Kreyenberg H, Hoelle W, Dueckers G, Handgretinger R, Lang P, et al. Increasing mixed chimerism is an important prognostic factor for unfavorable outcome in children with acute lymphoblastic leukemia after allogeneic stem-cell transplantation: possible role for pre-emptive immunotherapy? J Clin Oncol. 2004;22(9):1696-705.
