# Supplementary material for: Phase I/II study of the deacetylase inhibitor panobinostat after allogeneic stem cell transplantation in patients with high-risk MDS or AML (PANOBEST trial)
Source: Leukemia. 2017 Sep 1;31(11):2523–5. doi: 10.1038/leu.2017.242 (PMC5668491; doi:10.1038/leu.2017.242)
Supplement: Supplementary Figure Legend S2 [file leu2017242x7.docx]

**Figure legend S2**: **Patient allocation to administration schedule and dose levels.** The first group of 12 patients (cohorts 1-3) received panobinostat weekly at dose levels of 10, 20 and 30 mg TIW. Patients in cohorts 4-6 received panobinostat during alternating weeks at dose levels of 20, 30 and 40 mg TIW. In the expansion cohorts 7A and 7B, an additional 9 patients in each schedule received panobinostat at the respective MTD.
