# Supplementary material for: Phase I/II study of the deacetylase inhibitor panobinostat after allogeneic stem cell transplantation in patients with high-risk MDS or AML (PANOBEST trial)
Source: Leukemia. 2017 Sep 1;31(11):2523–5. doi: 10.1038/leu.2017.242 (PMC5668491; doi:10.1038/leu.2017.242)
Supplement: Supplementary Figure Legend S3 [file leu2017242x8.docx]

**Figure legend S3**: **Reconstitution of immune cells**

Methods

Flow cytometric analysis was performed to determine cellular immune reconstitution post HSCT. Peripheral blood (PB) EDTA samples were prospectively collected and analyzed within a maximum of 24 hours using a FC500 5-color flow cytometer (Beckman Coulter, Krefeld, Germany). Absolute cell count was calculated from the percentage values using a dual-platform approach. Monoclonal IgG1 and ^2^IgG2a antibodies against CD45, CD3, CD4, CD8, CD19, CD56, ^2^CD14, CD25^#^ and CD127 (^#^ from Becton Dickinson (BD), Heidelberg, Germany, all others from Beckman Coulter, Immunotech, Marseille, France) were conjugated with fluorescein isothiocyanate (FITC), phycoerythrin (PE), phycoerythrin-Texas-Red (ECD), phycoerythrin-cyanine-5 (PC5) and phycoerythrin-cyanine-7 (PC7) for staining. An automated lyse/no wash procedure with a fixation step (TQ-Prep^TM^ Workstation, Beckman Coulter, Krefeld, Germany) was performed. The first and second tube examined the quantity of leukocytes, lymphocytes, CD14^+^ monocytes, CD3^+^ T cells, CD3^+^CD4^+^ helper T cells, CD3^+^CD8^+^ cytotoxic T cells, CD56^+^CD3^-^ NK cells and CD19^+^ B cells. The third panel allowed the measurement of the CD4^+^CD25^++^CD127^dim/neg^ regulatory T cell amount.

Statistics

Longitudinal analysis of cellular reconstitution was performed using the R package *Linear and Nonlinear Mixed Effects Models* (nlme) (1). Cell counts were logarithmically transformed in order to attain residuals with normal distribution. Mixed-effects linear regression models were fitted for each cell population considering repeated measurements for individual patients. The significance level was not adjusted for multiple testing. P-values were considered significant for p<0.05 (*) and p<0.01 (**).

Results

Data is available from 26 out of 42 patients (62%) during study treatment; the number of patient samples available for each point is indicated by schedule. CD8^+^ T cell counts **(Figure S2A)** and CD4^+^ T cell counts (**2B)** did not differ between study schedules. The absolute number of regulatory T cells was not significantly different between schedules **(2C),** whereas the frequency of regulatory T cells (Treg) was significantly higher in patients treated according to schedule B **(2D)**. NK and B cell recovery are depicted in **2E** and **2F**, respectively. The grey area symbolizes reference data of Comans-Bitter et al. (2) (A,B,E,F) and Schatorjé et al. (3) (C,D) presenting the 5^th^ and 95^th^ percentile of healthy adults.

References

1. Pinheiro J, Bates D, DebRoy S, Sarkar D, Team RC. nlme: Linear and Nonlinear Mixed Effects Models. R package version 3.2.1 ed2015.

2. Comans-Bitter WM, de Groot R, van den Beemd R, Neijens HJ, Hop WC, Groeneveld K, et al. Immunophenotyping of blood lymphocytes in childhood. Reference values for lymphocyte subpopulations. J Pediatr. 1997;130(3):388-93.

3. Schatorje EJ, Gemen EF, Driessen GJ, Leuvenink J, van Hout RW, van der Burg M, et al. Age-matched reference values for B-lymphocyte subpopulations and CVID classifications in children. Scand J Immunol. 2011;74(5):502-10.
